# Improving Social Odometry Robot Networks with Distributed Reputation Systems for Collaborative Purposes

**DOI:** 10.3390/s111211372

**Published:** 2011-11-30

**Authors:** David Fraga, Álvaro Gutiérrez, Juan Carlos Vallejo, Alexandre Campo, Zorana Bankovic

**Affiliations:** 1 Departamento de Ingeniería electrónica, ETSI Telecomunicación, Universidad Politécnica de Madrid, Av. Complutense, 30, 28040 Madrid, Spain; E-Mails: jcvallejo@die.upm.es (J.C.V.); zorana@die.upm.es (Z.B.); 2 Tecnologías Especiales Aplicadas a la Telecomunicación, ETSI Telecomunicación, Universidad Politécnica de Madrid, Av. Complutense, 30, 28040 Madrid, Spain; E-Mail: aguti@etsit.upm.es; 3 IRIDIA, CoDE, Université Libre de Bruxelles, Av. F. Roosevelt, 50, CP 194/6, 1050 Brussels, Belgium; E-Mail: alexandre.campo@ulb.ac.be

**Keywords:** collaborative robots, robot networks, social odometry, collective decision, reputation systems, trust algorithms, unsupervised techniques

## Abstract

The improvement of odometry systems in collaborative robotics remains an important challenge for several applications. Social odometry is a social technique which confers the robots the possibility to learn from the others. This paper analyzes social odometry and proposes and follows a methodology to improve its behavior based on cooperative reputation systems. We also provide a reference implementation that allows us to compare the performance of the proposed solution in highly dynamic environments with the performance of standard social odometry techniques. Simulation results quantitatively show the benefits of this collaborative approach that allows us to achieve better performances than social odometry.

## 1. Introduction

This paper proposes a new approach for collaborating purposes in a swarm of robots working together to achieve a goal. Robots are individual sensors highly efficient, equipped with sufficient abilities, that can be exploited jointly. The collaborative swarm is a group of entities that work together to achieve a common objective. They make intelligent decisions to achieve a foraging goal which requires some mechanism of collaboration by means of social odometry. In social odometry, each robot is a sensor for the other robots of the swarm. The importance of social odometry lies on the fact that the swarm (the collectivity) allows the robots to collaborate to achieve a common objective because the individuals are working together.

Many robotics applications require the robots to be localized to achieve different tasks. Different solutions to the localization problem have been implemented. Among these, odometry is probably the most used as it provides easy and cheap real time position information by the integration of incremental motion information over time. Unfortunately, this integration causes an accumulation of errors during the movement of the robot, and this can be a great drawback in some robotic applications, such as foraging, where the robots have to find, select and exploit resources from unknown locations.

Different approaches have been implemented to deal with this complexity; however, those solutions have a number of different limitations: (i) they are power consuming in terms of computation [1,2]; (ii) some robots are not allowed to move or they have its mobility limited [3]; (iii) robots must maintain visual contact at all times with the rest of the group [4]; and (iv) in some cases robots have to communicate with a central device to update or download maps of their environment, synchronize movements, or update positions [5].

Social odometry [6,7] is a novel solution that exploits self-organized cooperation in a group of robots to reduce each individual location error. Each robot location knowledge consists of an estimate of its own location and an associated confidence level that decreases with the distance traveled since the last known location. In order to maximize its confidence about its estimate, each individual tries to update it by using the information available in its neighborhood. Estimated locations, confidence levels and actual locations of the robots co-evolve in parallel in order to guide each robot to the correct objective.

In this paper, we work with a classical swarm foraging scenario: a number of resource items (usually called “prey”) are randomly scattered in the arena. In this context, robots search and retrieve those resource-items back to a specific place (usually called “nest”). The performance of the robot network in this kind of foraging systems can be measured as either the resources-items collected by unit of time, or the time robots need to exhaust the resources.

As aforementioned, social odometry uses a simple reputation system based on the distance traveled. However, from the point of view of reputation systems techniques, foraging scenarios have more useful trust information sources that have not been used in previous works [6,7].

In this paper, we state that by defining a complete architecture and following a systematic reputation-system analysis and design processes, it is possible to improve the performance of social odometry. Hence, we propose a complete reputation-system architecture and an analysis and design methodology, and provide a reference implementation that allow us to compare the performance of the proposed approach with the performance of social odometry.

The rest of this paper is organized as follows: Section 2 explains how social odometry works. In Section 3 we provide a brief introduction to the main topics related to reputation systems and provide a reference architecture. Section 4 analyzes in detail how reputation systems can improve social odometry robot networks. In Section 5 we present the experimental results. Finally, in Section 6 we draw some conclusions.

## 2. Social Odometry

### 2.1. The Odometry Problem

Odometry is probably the most used localization method. It provides easy and cheap real time position information through the integration of incremental motion information over time without the need for any other device. In all odometry techniques, a travel path is derived from sensors computing the movement of the robot. However, the accuracy of odometry measurements strongly depends on the kinematics of the robot. Unfortunately, because of the integration of the robot’s movement, odometry calculation causes an accumulation of errors, where problems such as slippage, misalignment of the wheels or several other inaccuracies must be taken into account [8].

Odometry errors can be classified as either systematic or non-systematic errors [9]. Systematic errors can be modeled and corrected, while the non-systematic ones cannot be corrected and many classical techniques have been implemented to cope with them.

### 2.2. Learning from Others

Social odometry is a previously defined technique [7,10] which is not based on any map-like algorithm, and despite being inspired by the Kalman Filter [6,11], it does not require any explicit model of the movement errors. On the contrary, a relationship between the distance traveled and a confidence level allows the robots to select the closest resource site on a foraging-like scenario.

The key aspect of social odometry is that robots within the swarm act as virtual landmarks to the others and exchange their knowledge about the position of goal areas. Nonetheless, they have to deal with two main issues: (i) the robots only know estimated locations, not the real locations; and (ii) the more the robots travel the worse those estimates are.

To comply with the aforementioned characteristics, social odometry uses a range and bearing communication sensor [12,13] which provides a local, distributed and situated communication. This sensor allows the robots to obtain the information transmitted by their neighbors, as well as the range and bearing to the emitting source. The communication does not rely on any central unit. Moreover, no synchronization is needed by the robots to exchange their information, removing the need for a common time axis. However, because the robots do not have any inertial system, the sole common coordinate system lies on the range and bearing communication system.

### 2.3. Social Odometry Equations

In social odometry, we define the state vector of the robot *i* at time *k* as:

(1) $$x_{k}^{i}={\left[x_{k}^{i}\;\;y_{k}^{i}\;\;\theta_{k}^{i}\right]}^{T}$$

where $x_{k}^{i}$ and $y_{k}^{i}$ are the robot’s Cartesian coordinates and $\theta_{k}^{i}$ its orientation.

Moreover, the inverse of the confidence level $\left(p_{k}^{i}\right)$ is defined as distance travelled by the robot $\left(d_{k}^{i}\right)$.

Every robot keeps track of its movements and updates its *a priori* estimated location and confidence level about the different goals (*i.e.*, nest and prey) as:

(2) $$\begin{array}{l} {\hat{x}}_{k|k-1}^{\mathrm{goal},i}={\hat{x}}_{k-1|k-1}^{\mathrm{goal},i}+\Delta {\hat{x}}_{k}^{i}\\ p_{k|k-1}^{\mathrm{goal},i}=p_{k-1|k-1}^{\mathrm{goal},i}+\Delta d_{k}^{i} \end{array}$$

where $\Delta {\hat{x}}_{k}^{i}$ is the state vector displacement in the time step duration and $\Delta d_{k}^{i}$ is the distance travelled in the time step duration.

If there is no encounter between the robots, the *a posteriori* values are matched to the *a priori* values $\left({\hat{x}}_{k|k}^{\mathrm{goal},i}={\hat{x}}_{k|k-1}^{\mathrm{goal},i},p_{k|k}^{\mathrm{goal},i}=p_{k|k-1}^{\mathrm{goal},i}\right)$. Therefore, the confidence level decreases indefinitely. On the other hand, if two robots meet, the robots exchange information about their position and confidence level. In order to produce an *a posteriori* estimated location, each robot takes into account all information available, but weighs its sources in a different way:

(3) $${\hat{x}}_{k|k}^{\mathrm{goal},i}=\left(1-g_{k}^{\mathrm{goal},i}\right)\;{\hat{x}}_{k|k-1}^{\mathrm{goal},i}+g_{k}^{\mathrm{goal},i}\;\left({\hat{x}}_{k|k-1}^{\mathrm{goal},j}+x_{k}^{\mathrm{ij}}\right)$$

(4) $$p_{k|k}^{\mathrm{goal},i}=\left(1-g_{k}^{\mathrm{goal},i}\right)\;p_{k|k-1}^{\mathrm{goal},i}+g_{k}^{\mathrm{goal},i}p_{k|k-1}^{\mathrm{goal},j}$$

where $x_{k}^{\mathrm{ij}}$ is the vector from one robot *i* to robot *j* and *g_k_* represents the so-called pairwise comparison rule often adopted in evolutionary/social dynamics studies [14], to code the social learning dynamics, which makes use of the Fermi distribution:

(5) $$g_{k}^{\mathrm{goal},i}=\frac{1}{1+e^{-\beta \left(\Delta p_{k|k-1}^{\mathrm{goal},ij}\right)}}$$

where $\Delta p_{k|k-1}^{\mathrm{goal},ij}=p_{k|k-1}^{\mathrm{goal},i}-p_{k|k-1}^{\mathrm{goal},j}$ and *β* measures the importance of the relative confidence levels in the decision making.

Therefore, social odometry fuses the robot estimations based on their confidence levels. An exhaustive revision of the social odometry equations can be found in [11].

## 3. Reputation Systems

Trust and reputation have recently been suggested as an effective security mechanism for open and distributed environments (*Ad Hoc* networks, WSNs, P2P networks, *etc*.). Extensive research has been done on modeling and managing trust and reputation. Specifically, it has been demonstrated that rating trust and reputation of individual nodes is an effective approach in distributed environments not only to improve security, but to support decision-making and promote node collaboration.

There are many different definitions of trust and reputation [15]. In essence trust is a belief about future behavior that one participant in the system holds in others and it is based on its own experience, thus its main characteristic is subjectivity. On the other hand, reputation is considered to be the global perception of the behavior of a node based on the trust that others hold in it. Thus, reputation is considered to be objective.

**Figure 1.**
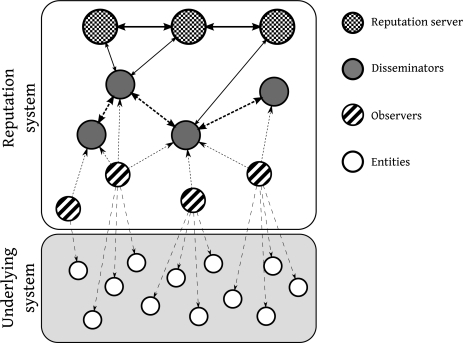
Generic reputation system architecture.

In order to identify the fundamental entities of reputation systems, an architectural model for reputation systems is presented (see Figure 1). Therefore, we can analyze all the entities involved in a trust/reputation dynamic and all the processes needed to effectively take advantage of this kind of systems.

- **Underlying System.** Reputation systems exist to improve the performance of another system in a specific way. This system is called *underlying system* and its basic components are called *entities*.
- **Observers.** They are the basic agents of the reputation system. They create and manage the trust used by the whole system.
- **Trust Information Sources.** In order to create a useful value of trust for entities, observers can use any of these sources: they can obtain information by direct observation of the *real world*; they can use their *memory*, so they are able to evaluate the historical behavior of the entities; they can use information provided by other *observers* (*communication*); they can use *categorization* as trust source information when the group the entities belong to is associated to a specific trust environment (this is very common in social interactions where it is called *prejudices*); and finally they can use the *reputation* value of the entities (this is common in early interactions or when the global perception of an entity is more important than the local perception).
- **Trust Algorithm.** In order to create a useful value of trust, observers process all or some of the aforementioned sources of information with an internal algorithm. This is a key element in the whole reputation system so it has to be chosen very carefully as we will see in the next sections.
- **Disseminators.** Trust information calculated by *observers* can be used by other observers or can be used to calculate reputation values. In order to allow this transmission of information, some agents within the reputation system can have the capacity of relaying trust information messages.
- **Dissemination Protocol.** Transmission of trust and reputation information carried out by the *disseminators* is based on the existence of a specific communication protocol that is commonly called *dissemination protocol*.
- **Reputation Servers.** Some special agents in the reputation system (or even none or all the agents) can use the trust information generated and distributed by the *observers* and *disseminators* to generate values of *reputation* for all the entities.
- **Reputation Algorithm.** In order to create a useful value of reputation, *reputation servers* use an internal algorithm.

Besides of analyzing these architectural elements, we should take into account how the reputation system is conditioned by the underlying system.

- **Topology.** Related to the dissemination protocol we find that the topology of the underlying system is a key factor. We can find as many topologies as in a generic distributed system (e.g., client-server, multi-agent systems, ad-hoc networks).
- **Timing.** Trust information acquisition, calculation or dissemination are vital processes. The moment *when* they happen can modify and determine the features and effectiveness of the reputation system. The three basic timing schemas are: periodic, event oriented and periodic adaptive.
- **Limitations of the Underlying System.** We must take into account all possible limitations that the underlying system can impose. Among others: communication or computational resources, storage capacity, power consumption, *etc*.
- **Requirements and Goals of the Underlying system.** Reputation systems are a way to improve an underlying system performance in a number of specific criteria. So, the most important task we have to carry out is to identify all these requirements and goals.

## 4. Reputation Systems in a Social Odometry Context

Social odometry exploits self-organized cooperation in a group of robots to reduce each individual location error using a simple and low-resources-consumption model. This allows us to use this localization technique in a wide range of real-life scenarios. If we could minimize this location error without increasing the complexity order of the solution, we would be able to both improve the performance of social-odometry applications and broaden even more the range of the systems where we can apply social odometry techniques.

As we described before, in order to improve the behavior of basic social odometry techniques we only have to analyze them from the reputation system point of view. In this section we propose and follow a methodology to analyze and design our reputation system.

It is based on three main steps: analyze the underlying system, identify the elements that are going to be part of the reputation system architecture and define how trust and reputation processes (algorithms) are going to be carried out by the system.

### 4.1. Underlying System Analysis

Based on the aforementioned structure we can identify the following topics about the underlying system.

- **Description of the Underlying System.** Based on a classical social odometry swarm behavior [7], we propose a richer and more complex scenario so we can analyze the viability of this solution in real-life environments. The additional features are: (i) **there are different models of robots** and it is well-known that they have different location performances (some models are better than others); (ii) within a specific model, **individual robots have different location performances** (but this specific performance is not known by the other robots).
- **Requirements and Goals.** Robots have to go to the source of resources (“prey”) and go back to the “nest” as many times as they can. This is a paradigmatic example of a *Maximization of the System Performance* scenario.
- **Topology.** There are not central services. The robots have full freedom of movements and all P2P communications between them are allowed if they are near enough.
- **Timing.** There is not a global clock to trigger whole-system behaviors. So the system is event oriented.
- **Limitations.** The main limitations are based on the communication, computational and storage resources of the robots. Power consumption might be a limitation too, but we will not take it into account in this paper.

### 4.2. Reputation System Analysis

If we review the elements and processes of the proposed reputation system architecture, we can identify the following ones:

- **Observers.** Every robot in the underlying system is a sensor in the network, so it can be an observer in the reputation system.
- **Trust Information Sources.** The main disadvantage of the previous social odometry approach is that it misses some of the traditional trust information sources. They use information from the *real world* (obtained by their sensors) and information from other observers in a simple way (in the P2P robot-to-robot *communications*), however they lack for an accurate use of *memory* and *categorization*. On the one hand, *memory* is a key factor in the system. In the basic social odometry scenario robots only *remember* how long they have been walking since they found a known location. However, a model with more historical information could improve the precision of any trust algorithm. We will see how simple concepts like the global performance of the robot (total distance/number of locations found or number of round-trips done) can significantly increase the throughput of the system. On the other hand, the use of *categorization* can help us to improve the behavior of the system in the early stages. Therefore, robots can have a more accurate knowledge of the confidence level of the positions transmitted by other robots. Even when they have not already had a minimum amount of historical information (*memory*).
- **Trust Algorithm.** Because of the special importance of this matter it will be discussed in detail in the next subsection.
- **Disseminators.** Every robot in the underlying system can act as a disseminator in the reputation system. *Communication* is essential in the social odometry and we will take advantage of it.
- **Dissemination Protocol.** All communications in the system are robot-to-robot communications so we do not need a complex protocol. We only have to deal with physical and link layer issues. Network layer features are not needed.
- **Reputation Server.** Because of the topology of the underlying system and its limitations, there are not any global services, so we will not have a reputation server for the whole system. We could evaluate if all robots or some of them could act as reputation servers, however the concept of reputation would not be realistic in the defined scenario because in this kind of swarms there is not any kind of *a priori* individual knowledge. Besides, we do not have an efficient mechanism to propagate information throughout the network. So, we could not disseminate the reputation values. Anyway, future works could deal with this idea of introducing reputation servers within the system and analyzing advantages and drawbacks of this proposal.
- **Reputation Algorithm.** Based on the previous point, a reputation algorithm is not needed in this scenario.

### 4.3. The Trust Algorithm. Conceptual Approach and Trust Sources

Based on previous works in social odometry and reputation systems, we will try to define the main requirements of our trust algorithm.

In a system composed of entities with different performance levels, the possibility of having an *a priori* knowledge of this performance or a knowledge of the predictable behavior of these entities can help us to improve the global performance. Optimal filters are a classical approach to this topic, but they are computational expensive compared to the resources available in the robots [16]. However, in a reputation system world, this kind of knowledge is often modeled in a more simple way: the concept of *category*.

Moreover, we have identified that the number of round trips divided by the distance traveled can be a good estimator of the individual performance of every robot in the system.

Finally, the information exchange carried out by the social odometry approach has proved to be valid in this kind of environments. However, it is limited to the transmission of *personal* information. As mentioned before, one of the main trust information sources is carried out by the disseminators, so besides transmitting their own location information, they could transmit trust information about previous known robots based on its individual performances. In this way, trust information could be disseminated faster and the whole system performance might be improved as well.

Based on these previous ideas our trust algorithm will be defined as follows:

The inputs for our algorithm will be: (i) the distance traveled since the last known location, so we can keep the advantages of the classical social odometry approach; (ii) the category or type of robots in the system, so we can introduce an *a priori* knowledge but in a simpler way than using other common techniques (such as Kalman filters [17]); and (iii) the ratio distance divided by number of round-trips, so we will have an estimate of the individual performance.

Moreover, we will store these inputs so we can use this historical information. Finally, we will promote the trust dissemination between robots.

### 4.4. The Trust Algorithm: Algorithm Specification

In order to implement the algorithm we could have used some standard trust algorithm, such as beta algorithm [18], genetic algorithms [19] or self-organized maps [20]. However, in this environment none of them suits our requirements. They are computational expensive, so we decided to adapt the Fermi distribution used in social odometry.

Based on this equation, we are going to introduce the main improvements we commented before. First of all, we will introduce the idea of *category*. In our system there will be three kinds of robots based on the accuracy of their location sensors. Respectively, tolerance will be 2%, 5% and 10%. To introduce this concept in the algorithm we will model this tolerance as maximum errors, so the new “confidence level” will be weighted by this error estimation:

(6) $$E_{\mathrm{category},j}=\{\begin{array}{l} 0.02,\;\;\;\text{if}\;\text{tolerance}\;\text{is}\;\pm 2\%\\ 0.05,\;\;\;\text{if}\;\text{tolerance}\;\text{is}\;\pm 5\%\\ 0.10,\;\;\;\text{if}\;\text{tolerance}\;\text{is}\;\pm 10\% \end{array}$$

(7) $${{ɛ}^{\prime }}_{j}={ɛ}_{j}*E_{\mathrm{category},j}\frac{1}{d_{j}(\mathrm{loc})}\left(1-E_{\mathrm{category},j}\right)$$

The next step is to introduce the idea of *memory* in the form of an estimated error. We will use the aforementioned simple ratio: total distance divided by number of round-trips.

Firstly, we define “estimated distance from nest to prey” for an entity *i* as follows:

(8) $$D_{NP,i}=\frac{T_{\mathrm{length},i}}{N_{\mathrm{rounds},i}}$$

So, the better the performance the shorter the distance.

Then, we define the estimated error of the entity *j* (observee) from the point of view of the entity *i* (observer) as given by the next equation:

(9) $$E_{\mathrm{memory},ji}=\{\begin{array}{ll} \frac{D_{N\;P,j}-D_{N\;P,i}}{D_{N\;P,i}}, & \text{if}\;D_{N\;P,j}-D_{N\;P,i}>0\\ 0, & \text{if}\;D_{N\;P,j}-D_{N\;P,i}\leq 0 \end{array}$$

There are two important ideas we should clarify. Firstly, we have introduced the idea of subjectivity. We remarked “trust” is a subjective concept but we had not yet used this fact: the “confidence level” now depends on the observer. Secondly, related to the Equation (9), we only define *E_memory,ji_* ≠ = 0 when the observer has a better performance than the observee. In this way, robots with worse performance cannot say that robots with better individual performances are wrong.

Finally, we can introduce this memory error ratio in our “confidence level” as follows:

(10) $${ɛ}_{j,i}^{\prime \prime }={ɛ}_{j}^{\prime }*E_{\mathrm{memory},ji}=\frac{1}{d_{j}(\mathrm{loc})}\left(1-E_{\mathrm{category},j}\right)\left(1-E_{\mathrm{memory},ji}\right)$$

Finally, the dissemination process does not need to be introduced in the algorithm, but in the exchanged information. If robots exchange their *D_NP_* tables and their estimates of different locations, an entity can use those estimates even when it has not had a previous direct communication with other entities. However, this can introduce a significant overload both in storage and computational resources. We will analyze the effects of the trust dissemination in the next section.

## 5. Experimental Results

### 5.1. Simulation Tools

The proposed algorithms have been tested in simulation. We used a simulator of robot networks developed by the IRIDIA research group from Université Libre de Bruxelles. This simulation platform is a fast multi-robot simulator for the e-puck robot [21,22]. It has a custom rigid body physics engine, specialized to simulate only the dynamics in environments containing flat terrain, walls and holes. This restriction allows for certain optimization in the computation of the physics and, thereby, reduces the computational resources needed for running simulations (see [23] for more details). This platform has been combined with a high level abstraction layer based on a reputation-system simulator called TRS-SIM, designed and implemented by the DIE research group from Universidad Politécnica de Madrid. TRS-SIM is now under a final revision previous to its public release. However, it has already been successfully used in several scientific works [24–27] related to trust and reputation systems applied to different disciplines.

The robot network simulator is responsible for kinematic, sensing, decision making and communication tasks while the logic of the reputation system simulator is responsible for the trust generation and management and provides high level information for the decision-making module of the robot network simulator. The combination of these two specific simulators allow us to derive novel results in this area of knowledge.

In our simulations, a robot is modelled as a cylindrical body of 3.5 cm in radius that holds 8 infrared proximity sensors distributed around the body, 3 ground sensors on the lower-front part of the body and a range and bearing communication sensor. IR proximity sensors have a range of 5 cm, while the range and bearing sensor used for the communication has a range of 15 cm. For the three types of sensors, we have sampled real robot measurements and mapped the data into the simulator. Furthermore, we added uniformly distributed noise to the samples in order to simulate effectively the different sensors. Up to ±20% noise is added to the infrared sensors and up to ±30% to the ground sensors. In the range and bearing sensor, noise is added to the range (up to ±2.5 cm) and bearing (up to ±20) values. Moreover, each message emitted can be lost with a probability that varies linearly from 1% when the sender-receiver distance is less than 1 cm, to 50% when the two robots are 15 cm from each other. A differential drive system made up of two wheels is fixed to the body of the simulated robot. Errors have also been introduced into the encoder sensors chosen uniformly random in ±20% of the maximum movement at each time step for each wheel.

### 5.2. Simulation Experiment

In this section, we compare results obtained for different social odometry experiments with the ones obtained for the proposed reputation system scheme based on all the analysis and design decisions followed in the previous sections. Experiments have been tested in a typical foraging scenario. The selection of this scenario has been made in order to allow for comparison with previous social odometry experiments. However, an extension and generalization of the social odometry algorithms is suggested in Section 6.

It is important to notice that typical social odometry experiments assume that all the robots in the swarm are homogeneous. We have already defined in Section 3 that reputation systems are able to improve the swarm behavior even if the robots are heterogeneous (e.g., differences in the fabrication process). Therefore, all the experiments presented in this section assume the swarm is made up of three categories of robots related to the fabrication process.

Based on the previous assumptions different experiments have been implemented:

- **No Odometry Error**: robots in the swarm do not have odometry error. Therefore, they navigate with a precise knowledge about the goals location $\left({\hat{x}}_{k}^{\mathrm{goal},i}=x_{k}^{\mathrm{goal},i},p_{k}^{i}=0;\forall k,\;\;i\right)$
- **Homogeneous Covariance Knowledge**: robots implement a Kalman Filter to fuse their own information and the one provided by their neighbor. In these experiments, the robots need to calculate the Kalman gain every time step. Because of the comparison with previous works, all the robots assume they have the same noise on both the kinematic and communication for the Kalman Filter equations. Moreover, each robot transmits its estimated location and its own a posteriori covariance matrix when it meets with other neighbors.
- **Social Odometry**: robots communicate using the social odometry filter presented in Section 2.3. In these experiments the robots only transmit their estimated location and confidence level (inverse to the distance traveled).
- **Heterogeneous Covariance Knowledge**: robots uses a Kalman Filter to fuse their own information and the one provided by their neighbor. As in the homogeneous covariance knowledge, the robots need to calculate the Kalman gain every time step. In this experiment the estimated noise is based on the category of the robots involved.
- **Advanced Reputation System—Category**: robots use the proposed reputation system. The trust algorithm only uses the *category* improvement described before (based on the Equation (7)). They must transmit their estimated location, the confidence level and a value based on the quality of their fabrication process.
- **Advanced Reputation System—Memory**: robots use the proposed reputation system. The trust algorithm uses both *categorization* and memory as new improvements (based on the Equation (9)). Moreover, they transmit their estimated location, the confidence level, a value based on the quality of their fabrication process and an average value of reliability based on their previous performance.
- **Advanced Reputation System—Dissemination**: robots use the proposed reputation system and disseminate trust information to other robots. So, they transmit their estimated location, the confidence level, a value based on the quality of their fabrication process, an average value of reliability based on their previous performance and a set of average values based on previous communications with other robots.

Finally, the simulations were carried out in a 3 *×* 3 m ^2^ and a 5 *×* 5 m^2^ arenas with two marked areas (“prey” and “nest”), and 30 robots were involved in every experiment. To obtain significant statistical data, the simulations sets were performed one thousand times each.

### 5.3. Computation and Communication Complexity

#### 5.3.1. Computation Complexity

As aforementioned, covariance knowledge experiments make use of Kalman Filters. The covariance matrix ***P***_*k*|*k*−1_ is updated based on the previous *a posteriori* estimated covariance matrix (***P***_*k*−1|*k*−1_) and the noise ***v***_*k*−1_ through its covariance matrix ***Q***_*k*−1_:

(11) $${\hat{x}}_{k|k-1}=f\left({\hat{x}}_{k-1|k-1},u_{k-1},0\right)$$

(12) $$P_{k|k-1}=A_{k}P_{k-1|k-1}A_{k}^{T}+V_{k}Q_{k-1}V_{k}^{T}$$

where, ***A****_k_* and ***V****_k_* are the Jacobians of *f*(*·*) with regard to *x_k_* and *v_k_* respectively, and ***P***_0_ = 0.

On the other hand, in the social odometry, the prediction stage is directly related to the confidence level. Since the spectral norm of the covariance matrix ***P*** grows endlessly until a communication is established or the robots arrive at one of the goals, we define the inverse of the *a priori* confidence level $\left(p_{k|k-1}^{i}\right)$ of robot *i* as the distance travelled $\left(d_{k}^{i}\right)$ since the robot left a specific area. Therefore the prediction stage for the induced covariance matrix is defined as:

(13) $$p_{k|k-1}^{i}=d_{k}^{i}$$

This implementation allows the robot not to calculate the covariance matrix at each time step, and therefore to save computational time.

Moreover, in the covariance knowledge experiments, the correction stage transforms the *a priori* estimated state (*x̂*_*k*|*k*−1_) into the *a posteriori* estimated state *x̂*_*k*|*k*_. The *a posteriori* estimated state (*x̂*_*k*|*k*_) is adjusted in proportion to the Kalman gain (***K****_k_*), which specifies the degree to which the *a priori* estimation and the measurement *z_k_* are incorporated into the *a posteriori* state. Finally, the *a posteriori* covariance matrix ***P****_k|k_* is also adjusted based on the Kalman gain.

(14) $$K_{k}=P_{k|k-1}H_{k}^{T}{\left(H_{k}P_{k|k-1}H_{k}^{T}+W_{k}R_{k}W_{k}^{T}\right)}^{-1}$$

(15) $${\hat{x}}_{k|k}={\hat{x}}_{k|k-1}+K_{k}\left(z_{k}-h\left({\hat{x}}_{k|k-1},0\right)\right)$$

(16) $$P_{k|k}=\left(I-K_{k}H_{k}\right)P_{k|k-1}$$

where, ***H****_k_* and ***W****_k_* are the Jacobians of *h*(*·*) with regard to *x_k_* and *w_k_* respectively.

Once again, because of the simplification of the covariance knowledge on the social odometry experiments we define *g* as the scalar value representative to the Kalman gain:

(17) $$g_{k}^{i}=\frac{1}{1+e^{-\beta \left(\Delta p_{k|k-1}\right)}}$$

Hence, we use a weighed average to obtain the new location ${\hat{x}}_{k|k}^{i}$ and the inverse of the confidence level $p_{k|k}^{i}$ using the Fermi function:

(18) $${\hat{x}}_{k|k}^{i}=\left(1-g_{k}^{i}\right)\;{\hat{x}}_{k|k-1}^{i}+g_{k}^{i}\;\left({\hat{x}}_{k|k-1}^{j}+x_{k}^{\mathrm{ij}}\right)$$

(19) $$p_{k|k}^{i}=\left(1-g_{k}^{i}\right)\;p_{k|k-1}^{i}+g_{k}^{i}p_{k|k-1}^{j}$$

Therefore, it is observed that social odometry implementations are based on scalar values calculations, while covariance knowledge experiments make use of matrices.

#### 5.3.2. Communication Complexity

Because robots in our experiments are used as the measurement *z_k_* to correct the estimates, the estimated state and error needs to be transferred between the robots. In all experiments, robots transmit the *a priori* estimated state (*x̂*_*k*|*k*−1_), but differences come up with the estimated error communication. In the covariance knowledge experiments robots need to transmit the *a priori* covariance matrix (***P***_*k*|*k*−1_) while in the social odometry robots only transmit scalar values. Table 1 shows a comparison about the information transmitted between the individuals. A maximum of three scalar values is transmitted in all social odometry experiments, with the exception of the dissemination experiment, which depends on the size of the set which must be transmitted. However, as aforementioned, this increase in the communication load is balanced thanks to the reduction on the computation complexity.

### 5.4. Results and Discussion

**Figure 2.**
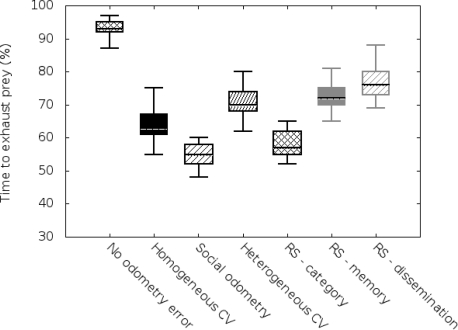
Simulation results for 3 *×* 3 m^2^ arena.

**Figure 3.**
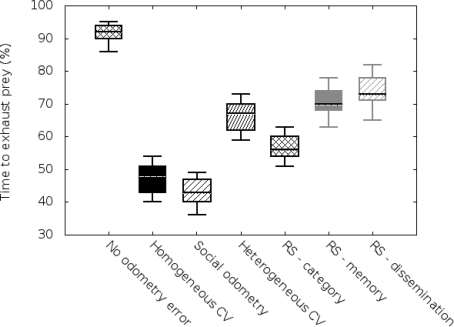
Simulation results for 5 *×* 5 m^2^ arena.

As mentioned before, we carried out two sets of simulations based on the size of the arena (3 × 3 m^2^ and 5 × 5 m^2^). We have implemented the same metric used previously in social odometry experiments, time to elapse the prey, in order to allow comparison with previous works. Results are compiled in Figures 2 and 3.

In the vertical axis we can see a value of performance, meaning by performance the time robots need to exhaust the resources in the “prey”. In order to visualize this ratio, we show it in percentage terms compared with the time robots, having no odometry errors, need to exhaust the “prey”.

On the other hand, in the horizontal axis, we will display a boxplot for each of the studied odometry techniques (no odometry errors, homogeneous covariance knowledge, basic social odometry, heterogeneous covariance knowledge, improved reputation model based on categorization, improved reputation model based on categorization and memory, and the complete proposed reputation model).

Results of the 3 × 3 m^2^ arena are shown in Figure 2. In this case, we can see the results obtained for the basic odometry scenario (no odometry errors, homogeneous covariance knowledge and social odometry) are similar to the results previously obtained in related works [7]. If we analyze the results with *category*-based-reputation system scenario (algorithm based in the Equation (7)), we can observe that the performance obtained in the basic social odometry experiment has been overcome. This difference is because category information helps robots to improve its coordination capabilities in the early stages of the simulation when the swarm is heterogeneous. However, we can see that the heterogeneous covariance knowledge performance has not been overcome by the *category*-based-reputation experiment. We should not forget that the social odometry approach is a simplification of the covariance knowledge methods.

Anyway, we can find the most important improvement when *memory* is considered and utilized as a trust information source (algorithm based in the Equation (9)). The main difference is because individual performance prevails over local situations (distance traveled since the last know location) and over general statements (categorization). This allow robots to trust more capable entities in the system and follow them as if they were “leaders”. In this case, the RS memory experiment shows a similar performance to the heterogeneous covariance knowledge (Wilcoxon test outputs *p* ≈ 0.5). It is important to say that this is because robots use more information than in the covariance approach but the improvement is compensated with the model simplification.

Finally, if we take advantage of the trust *dissemination* feature we notice that the results are better than in the heterogeneous covariance knowledge (*p* < 0.001 in the Wilcoxon test). This is because trust information is spread faster and the effect is similar to the use of categorization but with individual information: robots obtain an *a priori* information about the expected individual performance of other robots. Therefore, they can easily trust in the more capable individuals even without previous interactions. However, we have to remember that *dissemination* introduces a significant storage and computational resources overload. So we should evaluate robot’s resources in order to know if we can incorporate this technique to our robots.

If we compare these results with the results of the 5 × 5 m^2^ arena scenario (Figure 3), we can see that the reputation system approach offers even better performances. This is because the *a priori* knowledge (categorization) that the robots have helps them to improve their behavior in early stages and this effect is more important in wider scenarios. Without this *a priori* knowledge robots tend to randomly walk around longer throughout the arena and the global performance gets reduced.

Notice that all the experiments, making use of the reputation system, improve previous experiments done with social odometry. The main factor for this improvement is that the robots in the swarm have at hand more information than in standard social odometry algorithms. Therefore, the robots are able to generate a confidence level based not only on their own movement as in standard social odometry but also on the information provided by the other robots in the swarm integrated in time.

## 6. Conclusions

In this paper we have described how a reputation system can improve the performance of a complex and unsupervised scenario. In order to show it, we reviewed a novel odometry technique, social odometry, and we improved the coordination capabilities of this kind of robot networks designing a reputation system that takes advantage of all the significant information sources we can find in the system. We selected the most suitable trust algorithm and dissemination policies in order to minimize the throughput degradation that less capable robots can induce in the global behavior of the system.

To take advantage of reputation system features we showed the main ideas of a reputation system analysis and design methodology. This methodology is based on the identification of architectural entities, trust and reputation information sources, dissemination algorithms, functional and non functional requirements.

This analysis allowed us to choose the constitutive elements and the more suitable trust algorithms in order to improve the global behavior of a social odometry scenario. Simulation results quantitatively showed that the benefits of this approach were based on the use of *categorization*, *dissemination* and especially *memory*. Since, all of them allowed us to achieve better performances than classical odometry approaches. However, an important drawback could appear with the use of *dissemination*. It requires a significant computational and storage overload in the robots, and this fact can limit its utilization in some real-life scenarios where robots have very few resources. Nonetheless, the resources required during simulation are computationally comparable to the one of the heterogeneous covariance knowledge.

As future work we propose to analyze the viability of introducing a reputation server and a reputation dissemination mechanism within this kind of swarm scenarios. Moreover, a future extension of social odometry should lie on the implementation of general metrics which allow for comparison with other mathematically grounded methods in mobile robotics (e.g., absolute mean error). Besides, the foraging scenario should be generalized and metrics based on the movement error should be extracted. For its implementation an abstract model of the robot and a well-defined random walk algorithm should be extracted in order to allow a concrete comparison between these algorithms.

## Figures and Tables

**Table 1.** Information transmitted between the robots when encounter occurs. ${\hat{x}}_{k|k-1}^{i}$ is the *a priori* estimated state, $d_{k}^{i}$ is the inverse of the confidence level (distance traveled), $q_{k}^{i}$ is the associated quality to the fabrication process, ${\bar{r}}_{k}^{i}$ is the average value of reliability based on their previous performance and $r_{k}^{s}$ represents the set of average values based on previous communications with other robots.

**Experiment**	**Information transmitted**
Covariance knowledge	${\hat{x}}_{k|k-1}^{i}$, $P_{k|k-1}^{i}$
Social odometry	${\hat{x}}_{k|k-1}^{i}$, $d_{k}^{i}$
RS category	${\hat{x}}_{k|k-1}^{i}$, $d_{k}^{i}$, $q_{k}^{i}$
RS memory	${\hat{x}}_{k|k-1}^{i}$, $d_{k}^{i}$, $q_{k}^{i}$, ${\bar{r}}_{k}^{i}$
RS dissemination	${\hat{x}}_{k|k-1}^{i}$, $d_{k}^{i}$, $q_{k}^{i}$, $r_{k}^{s}$
